# Idiopathic sclerosing encapsulating peritonitis (or abdominal cocoon)

**DOI:** 10.1186/1471-2482-6-3

**Published:** 2006-02-13

**Authors:** Costas Serafimidis, Ioannis Katsarolis, Spyros Vernadakis, George Rallis, George Giannopoulos, Nikolaos Legakis, George Peros

**Affiliations:** 1 4^*th *^Department of Surgery, ATTIKON University General Hospital, Athens Greece, 1 Rimini Avenue, 12462 Haidari, Athens Greece; 2 4^*th *^Department of Internal Medicine, ATTIKON University General Hospital, Athens Greece, 1 Rimini Avenue, 12462 Haidari, Athens Greece

## Abstract

**Background:**

Idiopathic sclerosing encapsulating peritonitis (or abdominal cocoon) is a rare cause of small bowel obstruction, especially in adult population. Diagnosis is usually incidental at laparotomy. We discuss one such rare case, outlining the fact that an intra-operative surprise diagnosis could have been facilitated by previous investigations.

**Case presentation:**

A 56 year-old man presented in A&E department with small bowel ileus. He had a history of 6 similar episodes of small bowel obstruction in the past 4 years, which resolved with conservative treatment. Pre-operative work-up did not reveal any specific etiology. At laparotomy, a fibrous capsule was revealed, in which small bowel loops were encased, with the presence of interloop adhesions. A diagnosis of abdominal cocoon was established and extensive adhesiolysis was performed. The patient had an uneventful recovery and follow-up.

**Conclusion:**

Idiopathic sclerosing encapsulating peritonitis, although rare, may be the cause of a common surgical emergency such as small bowel ileus, especially in cases with attacks of non-strangulating obstruction in the same individual. A high index of clinical suspicion may be generated by the recurrent character of small bowel ileus combined with relevant imaging findings and lack of other plausible etiologies. Clinicians must rigorously pursue a preoperative diagnosis, as it may prevent a "surprise" upon laparotomy and result in proper management.

## Background

Sclerosing encapsulating peritonitis is a rare cause of small bowel obstruction, and can be classified as idiopathic or secondary (most importantly and most frequently due to chronic ambulatory peritoneal dialysis). The idiopathic form (also known as *abdominal cocoon*) was first described by Foo et al in 1978[1,2]. It affects mainly young females from tropical and subtropical regions, but adult case reports from temperate zones can be encountered in literature. It is characterized by a thick, fibrotic, cocoon-like membrane, partially or totally encasing the small bowel. Clinically, it presents with recurrent episodes of acute or subacute small bowel obstruction, weight loss, nausea and anorexia, and at times with a palpable abdominal mass[2]. Most cases are diagnosed incidentally at laparotomy, as in the case presented, although a preoperative diagnosis is purported feasible by a combination of barium follow-through (concertina pattern or cauliflower sign and delayed transit of contrast medium) and computed tomography of the abdomen (small bowel loops congregated to the center of the abdomen encased by a soft-tissue density mantle)[3,4]. However, preoperative diagnosis requires a high index of clinical suspicion. Surgery (membrane dissection and extensive adhesiolysis) is the treatment of choice, and there is usually no need for bowel loop resection, especially when a preoperative diagnosis is feasible. Resection of the bowel is unnecessary and it increases morbidity and mortality. Resection is indicated only if the bowel is non-viable. An excellent long-term postoperative prognosis is most of the times guaranteed[5]. Recently, in a series of 5 patients, in addition to adhesiolysis, small bowel intubation was performed with good results[6].

## Case presentation

**Figure 1:**
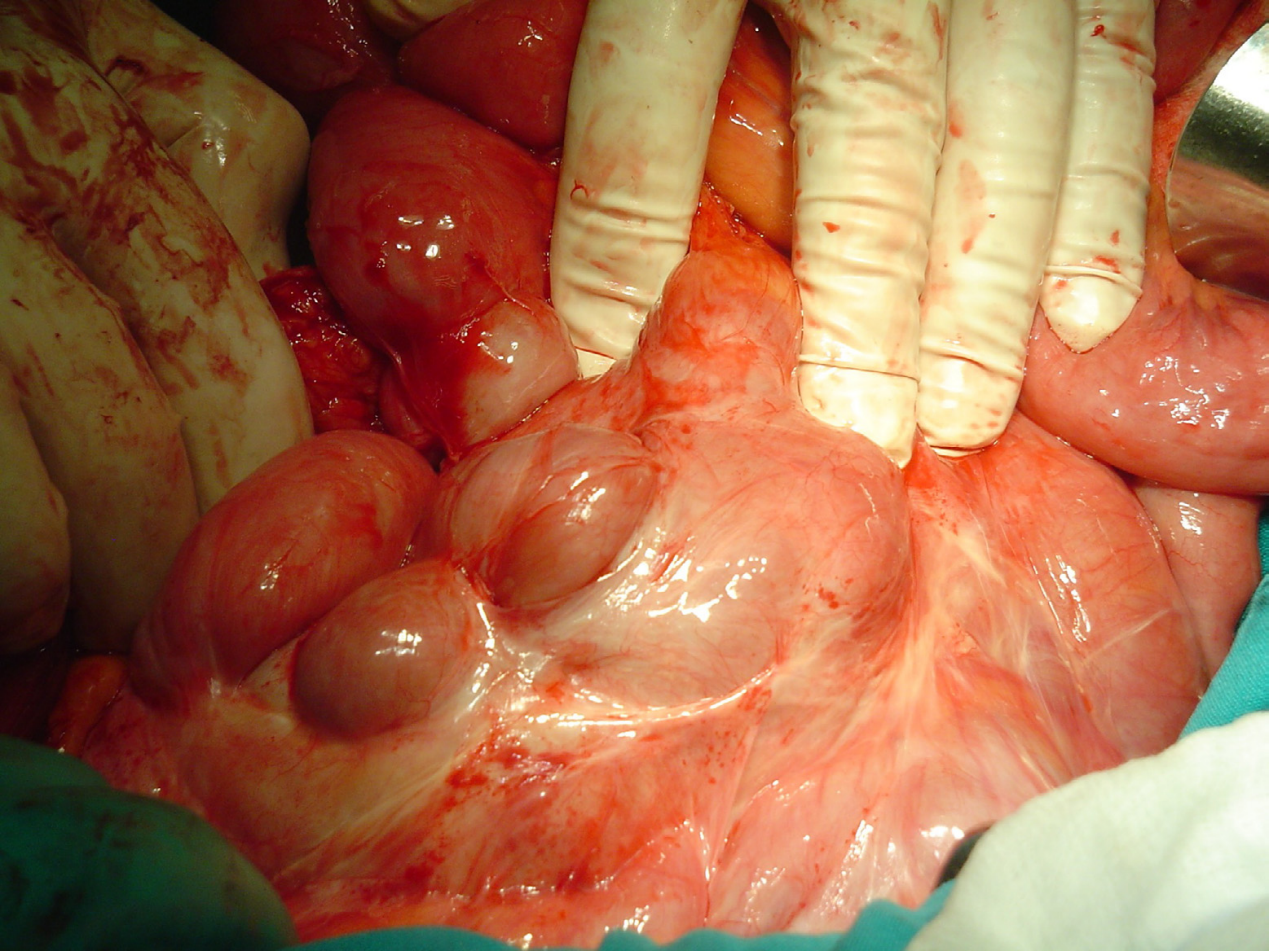
Intraoperative photograph: a fibrotic, cocoon-like membrane covering enteric loops.

**Figure 2:**
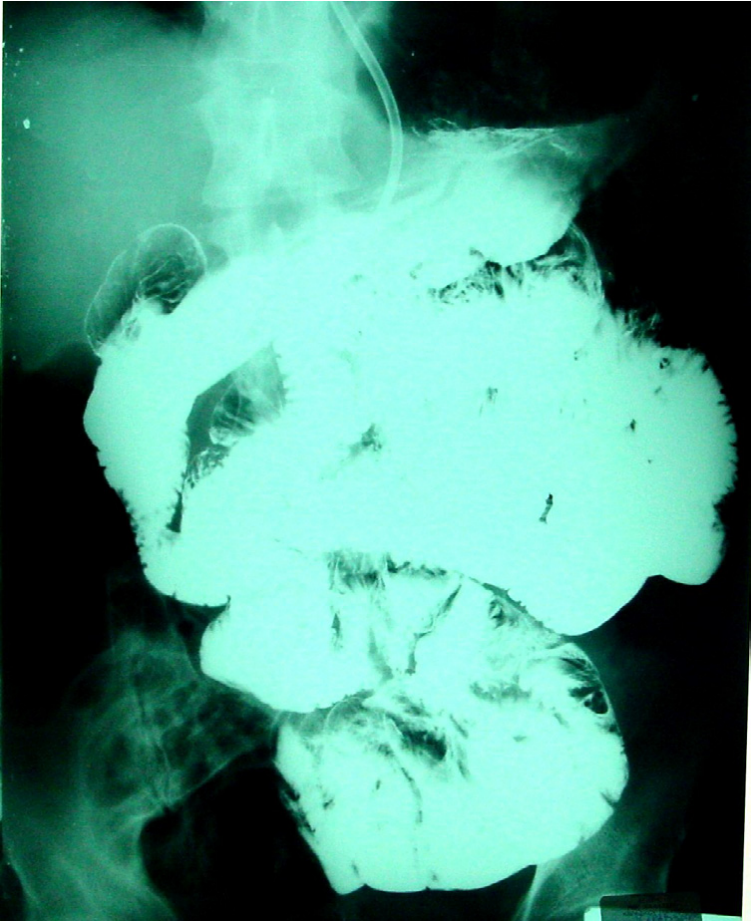
Small bowel follow-through examination performed on the first episode in another hospital. In the report, ileal loops are described as bunched and confined in the lower abdomen and pelvis apparently due to adhesions.

In September 2004, a 56 year-old man presented in A&E with 24-hour history of colicky abdominal pain and bilious vomiting. He reported 6 similar episodes, attributed to small bowel obstruction in the past 4 years, which required hospitalization and resolved with conservative treatment. He also admitted chronic constipation for the last 6 years, anorexia and 15 kg weight loss since his last admission (April 2004). He had no surgical or other medical history. On examination, he was in distress, but afebrile and haemodynamically stable. His abdomen was distended but non-tender, with increased bowel sounds in pitch and frequency. No palpable abdominal mass or organomegaly and no external hernias were present. Laboratory blood analyses were within normal limits. Plain abdominal X-ray showed few air-fluid levels centrally located, without free intraperitoneal gas. Ultrasound of the abdomen did not reveal any abnormalities. Contrast-enhanced abdomen computed tomography, performed on the same day, confirmed the diagnosis of small bowel ileus without providing any diagnostic clues. The patient was admitted to the general surgical ward and ileus resolved by day 3 conservatively. Endoscopy of upper and lower gastrointestinal tract and biopsies from the duodenum and colon provided normal findings. Although symptoms of obstruction had abated, the history of multiple relapses, the patient's complaints for poor quality of life and multiple admissions, as well as the undefined origin of the underlying pathology led to an exploratory laparotomy on day 7. On surgery, a fibrous capsule covering all the abdominal viscera was revealed, in which small bowel loops were encased, with the presence of interloop adhesions. The liver, stomach, appendix, right and left colon, as well as the sigmoid, were also covered and the greater omentum looked hypoplastic and encased in fibrous tissue (Figure 1). Incision of the thick membrane and extensive adhesiolysis of small bowel loops were performed without loop resection. Histology of the membrane showed thickened fibrocollagenous tissue without inflammation. A diagnosis of idiopathic sclerosing encapsulating peritonitis (*abdominal cocoon*) was established, due to intraoperative findings and by ruling-out any other condition explaining the patient's pathology. Postoperatively, a forgotten small bowel follow-through performed elsewhere in 2000 was brought to our attention by the patient, showing "*ileal loops bunched and confined in the lower abdomen and pelvis apparently due to adhesions*" (Figure 2). Postoperative recovery was uneventful and 10 months after the operation he is in good health.

## Conclusion

In the case presented, an intra-operative diagnosis of idiopathic sclerosing peritonitis was made in an adult male patient with a history of recurrent bouts of small bowel ileus. Pre-operative findings were inconclusive in his current admission. Although final surgical management would not have been modified, if results from imaging investigations during prior admissions were accessible in time, a pre-operative diagnosis could have been made possible.

Idiopathic sclerosing encapsulating peritonitis or *abdominal cocoon*, although a rare cause of a common surgical emergency such as small bowel ileus, may be responsible, especially in cases with recurrent attacks of non-strangulating obstruction in the same individual. A high index of clinical suspicion may be generated by the recurrent presentation of small bowel ileus combined with relevant imaging findings and lack of other etiologies Clinicians must rigorously pursue a preoperative diagnosis, as it may prevent a "surprise" upon laparotomy and unnecessary procedures for the patient, such as bowel resection.

## Competing interests

The author(s) declare that they have no competing interests.

## Authors' contributions

CS and IK designed the case report, researched the article and drafted the manuscript.

CS, SV, GR, GG, NL, GP carried out the surgery and were involved in all investigations.

All authors read and approved the final manuscript.

## Pre-publication history

The pre-publication history for this paper can be accessed here:
